# Oncogenic KRAS Regulates Secretion of Extracellular Vesicles and Surface Membrane Charge via Regulation of Phosphatidylserine in Pancreatic Cancer

**DOI:** 10.1021/acsnano.6c08263

**Published:** 2026-09-01

**Authors:** Paul A. Spezza, Kshipra S. Kapoor, Beatrice P. Pforr, Xin Luo, Kaira A. Church, Martin M. Bell, Bo Fan, Seoyun Kong, Elena V. Ramirez, Yi-Lin Chen, Fernanda G. Kugeratski, Sibani Lisa Biswal, Kathleen M. McAndrews, Florian Gebauer, Christoph Kahlert, Jacob T. Robinson, Raghu Kalluri

**Affiliations:** † Department of Cancer Biology, 4002 The University of Texas MD Anderson Cancer Center, Houston, Texas 77030, United States; ‡ Department of Bioengineering, 3990 Rice University, Houston, Texas 77005, United States; § Department of Electrical and Computer Engineering, Rice University, Houston, Texas 77005, United States; ∥ Department of Systems, Synthetic, and Physical Biology, Rice University, Houston, Texas 77005, United States; ⊥ Applied Physics Program, Rice University, Houston, Texas 77005, United States; # Department of Chemical and Biomolecular Engineering, Rice University, Houston, Texas 77005, United States; ¶ Department of Surgery II, University of Witten/Herdecke, Helios University Hospital Wuppertal, 42283 Wuppertal, Germany; ∇ Department of General, Visceral, and Transplant Surgery, Heidelberg University, Heidelberg University Hospital, 69120 Heidelberg, Germany; ○ Department of Neuroscience, Baylor College of Medicine, Houston, Texas 77030, United States; ⧫ Department of Pathology, The University of Texas Medical Branch, Galveston, Texas 77555, United States

**Keywords:** extracellular vesicles, microfluidic electrophoresis, zeta potential, pancreatic ductal adenocarcinoma, liquid biopsy

## Abstract

Extracellular vesicles (EVs) have the potential to be used as a liquid biopsy for cancer detection and treatment response assessment. Although the potential of EVs as disease-specific biomarkers has been promising, the rapid and specific enrichment of EVs from body fluids in a clinical setting remains challenging. To address this limitation, we developed a microfluidic electrophoresis (MEP) device for the label-free, charge-based enrichment of EVs from the serum of patients with pancreatic cancer (PaCa). We first validated the principle of charge-based cancer EV enrichment using cell-line-derived EVs, demonstrating that increased signaling through mutant Kirsten rat sarcoma viral oncogene homologue (KRAS), particularly Kras^G12D^, was associated with enhanced EV secretion and a shift toward more negative ζ-potential values. We then evaluated EVs derived from the serum of patients with PaCa to assess the clinical relevance and translational potential of the MEP platform. Further analyses identified phosphatidylserine and extraluminal DNA as part of the molecular determinants of the enhanced anionic nature of the PaCa-derived EVs. Overall, this proof-of-concept study introduces a promising microfluidic platform for enriching circulating cancer EVs with potential applications in the rapid detection of PaCa.

## Graphical abstract

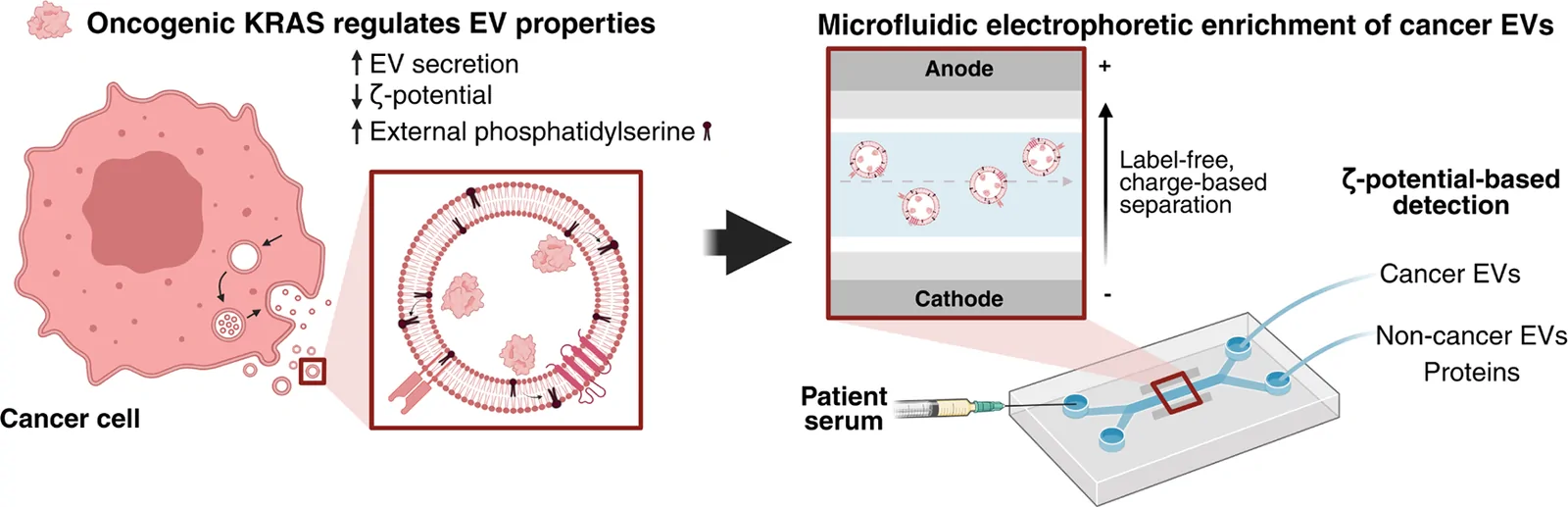

## Introduction

Pancreatic cancer (PaCa) is currently the fourth leading cause of death due to malignancy and is expected to be ranked second by 2030.^1,2^ About 90% of PaCa is pancreatic ductal adenocarcinoma (PDAC), for which Kirsten rat sarcoma viral oncogene homologue (KRAS) mutations are the primary drivers.^3^ KRAS mutations are frequently found in codon 12, with the most common being G12D, trailed by G12V.^4,5^ Lack of early detection, metastatic disease, and limited treatment options contribute to the high mortality rate of PaCa.^4–6^ Therefore, it is imperative that we attempt to develop rapid and sensitive strategies for screening PaCa patients at early stages with the potential for enhanced benefit from available treatments, thereby reducing PaCa-associated mortality.

An emerging approach for early diagnosis of PaCa is the use of liquid biopsies, which are easier to procure than tissue biopsies and can be collected using minimally invasive methods. The potential use of extracellular vesicles (EVs) isolated from patient serum as biomarkers for cancer liquid biopsy has recently attracted considerable attention.^7–13^ Exosomes (40–150 nm diameter), a subtype of EVs, are endosomal-derived vesicles with a phospholipid bilayer membrane that possess diverse molecular cargoes and mediate intercellular communication.^12,14–17^ Exosomes are of particular diagnostic interest because their contents may reflect disease-associated processes, including cancer progression and metastasis, altered cellular homeostasis, innate and adaptive immunity, and angiogenesis.^18–23^ Circulating EVs comprise both tumorigenic EVs and nontumorigenic EVs. Unfortunately, the latter can dilute the genomic assessment of EVs and can compromise sensitivity and specificity.^24^ Therefore, methods that enrich tumorigenic EVs to meet the detection threshold required by most downstream analyses are essential for facilitating the early detection of PaCa.^25^ However, selectively enriching cancer-derived EVs from serum or plasma remains challenging.^26,27^

Routinely used EV isolation techniques, including ultracentrifugation (UC), size exclusion, density gradient, and precipitation methods, primarily enrich or recover the total EV population from a sample based on physical properties such as size and density, rather than functional or biophysical heterogeneity.^28,29^ Alternative approaches that use silica substrates and antibody-coated beads to isolate EVs have been minimally explored.^30–32^ The antibody-coated bead assays involve multiple labeling steps, are time-consuming, and still cannot differentiate between tumorigenic and nontumorigenic EVs.^33,34^ Additionally, identifying critical molecular biomarkers on the EV surface that are specific to cancer cells remains a challenge.^32,35–37^ Such efforts have also been further hindered by EV heterogeneity within individual patient samples,^38,39^ which complicates the determination of biomarker specificity.

To address this challenge, we developed a label-free microfluidic electrophoresis (MEP) platform that utilizes an electric field to enrich EVs from the serum of PaCa patients based on differences in EV surface charge, as measured by zeta potential (ζ-potential), which we discovered to be a distinguishing hallmark of PaCa EVs. Additionally, we reported a link between oncogenic Kras expression, EV production, and a more negative EV ζ-potential. Furthermore, we discovered that extraluminal DNA and the anionic phospholipid phosphatidylserine (PS) on the EV surface contribute to the net negative ζ-potential of PaCa EVs, which is influenced by oncogenic Kras. Overall, these proof-of-concept results demonstrate that the MEP platform can be used as a tool for the enrichment of cancer EVs from the serum of patients with cancer, with potential for early detection of PaCa, by exploiting the anionic charge of PaCa EVs as a basic biophysical property.

## Results and Discussion

### Oncogenic KRAS Regulates EV Production and Net Charge

We evaluated the molecular and biophysical characteristics of EVs isolated from six different cell lines of pancreatic origin: human nonmalignant epithelial cell lines (HPNE and HPDE) and human malignant PDAC cell lines (PANC-1, T3M-4, MIA PaCa-2, and BxPC-3). Nanoparticle (NP) tracking analysis (NTA) showed that the isolated NPs had a size distribution typical of small EVs (Figure S1A). Cryogenic electron microscopy (Cryo-EM) analysis on both tumorigenic (PANC-1) and nontumorigenic (HPNE) EVs demonstrated that the isolated particles were enclosed by a lipid bilayer (Figure S1B). To further extend the characterization of EVs, we employed the bead-based flow cytometry approach to measure the expression levels of the tetraspanins, CD9, CD63, and CD81, which are commonly used as EV biomarkers, and demonstrated the presence of these markers on all the cell lines examined (Figure S1C).

EV surface charge was evaluated by measuring ζ-potential, an indicator of net membrane charge relative to the surrounding medium.^40^ Measurements were performed using a bulk ZetaSizer platform, providing robust ensemble averages suitable for assessing population-level charge differences relevant to charge-based separation approaches. In the nontumorigenic and tumorigenic pancreatic cell lines, we observed an association between more negative EV ζ-potential and increased aggressiveness of pancreatic cells (Figure 1A,B). The aggressiveness of the pancreatic cell lines was determined using a soft agar colony formation assay (Figure S1D) and associated with EV charge (Figure 1B). Since the mutant form of KRAS is the key driver of PaCa, we used ELISA to measure RAS activation in tumorigenic pancreatic cell lines (PANC-1 and BxPC-3) and a nontumorigenic pancreatic epithelial cell line (HPNE) and observed a strong inverse relationship (*R* ^2^ = 0.8788) between RAS activation and the ζ-potential of EVs derived from these cell lines (Figure 1C,D).

**Figure 1:**
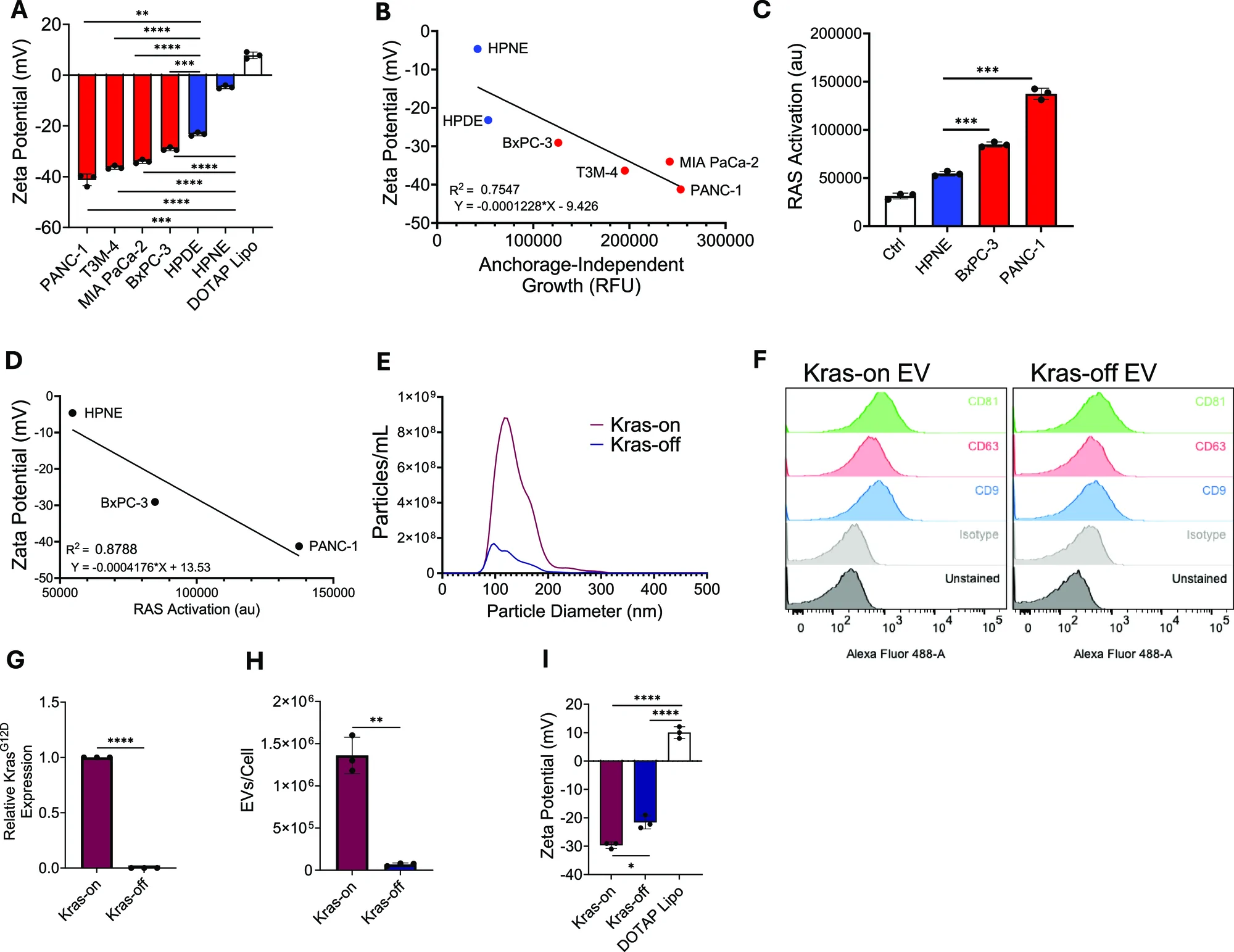
Oncogenic Kras regulates EV production and surface charge. (A) ζ-Potential of EVs released by the indicated tumorigenic and nontumorigenic pancreatic cell lines. (B) Relationship between EV ζ-potential and cellular aggressiveness, as measured by the soft agar colony formation assay. (C) RAS activation in the respective cell lines measured by ELISA. (D) Relationship between RAS activation and EV ζ-potential from respective cell lines. (E) Particle size and concentration of EVs produced by Kras-on and Kras-off cell lines. (F) Detection of surface markers CD9, CD63, and CD81 on EVs by flow cytometry in Kras-on and Kras-off cell lines. (G) Induction of Kras^G12D^ in the iKras cell line, confirmed by RT-qPCR. (H) EV production from Kras-on and Kras-off cell lines. (I) ζ-Potential of EVs from Kras-on and Kras-off cell lines. DOTAP liposomes used as a control. Data are represented as mean ± SD. Statistical significance was determined using unpaired *t*-test or linear regression (*p ≤ 0.05, **p ≤ 0.01, ***p ≤ 0.001, ****p ≤ 0.0001), *n* = 3 biological replicates.

To gather additional evidence that Ras activity might directly influence the negative surface charge of EVs, we utilized an inducible oncogenic Kras expression system (iKras cell line^41^) to evaluate the role of mutant Kras^G12D^ in EV secretion and contribution to EV surface charge (Figure 1E–I). Under the control of doxycycline, NTA analysis revealed that isolated EVs from both Kras^G12D^ On (Kras-on) and Kras^G12D^ Off (Kras-off) cells had a size distribution typical of small EVs and expressed canonical EV tetraspanins (Figures 1E,F and S1A). Induction of Kras^G12D^ was validated by RT-qPCR (Figure 1G), and Kras^G12D^ induction was associated with a significant increase in EV secretion (Figure 1H). Additionally, EVs derived from Kras-on cells were significantly more anionic than those derived from Kras-off cells (Figure 1I).

### Contribution of Distinct Biomolecules toward Net Charge of EVs

To explore the role of distinct biomolecules that could contribute to the charge of EVs, we devised approaches to evaluate how surface proteins, lipids, and extraluminal DNA contribute to the anionic charge of EVs.

To evaluate whether surface proteins are linked to the negative charge of EVs, we cleaved the extracellular domain of membrane proteins on the surface of EVs using Proteinase-K (Pro-K) (Figure 2A–D). NTA showed that Pro-K treatment of EVs did not induce major alterations in their size distribution or concentration (Figure 2A). Additionally, we tested whether Pro-K treatment was successful at digesting the majority of extracellular domains of membrane proteins using flow cytometry of bead-bound EVs. Using the tetraspanin CD81 as a readout, we observed that expression of CD81 was decreased upon Pro-K treatment compared to untreated EVs (Figure 2B). We also observed that Pro-K-treated and untreated EVs from both tumorigenic PANC-1 cells and nontumorigenic HPNE cells had equivalent negative charges with no significant difference in ζ-potential (Figure 2C). To determine the integrity of EVs after Pro-K treatment, we quantified the levels of protein localized in the lumen of EVs. We used single-particle flow cytometry to detect GFP in EVs derived from CD63-GFP-expressing PANC-1 cells, in which GFP is fused to the luminal domain of CD63 (Figure 2D). GFP levels of Pro-K-treated and untreated samples revealed no differences in GFP levels between the samples, supporting the fact that the integrity of the lipid bilayer was not disrupted (Figure 2D). Together, these findings suggest that the proteins on the EV surface are not the dominant determinant of ensemble EV surface charge.

**Figure 2:**
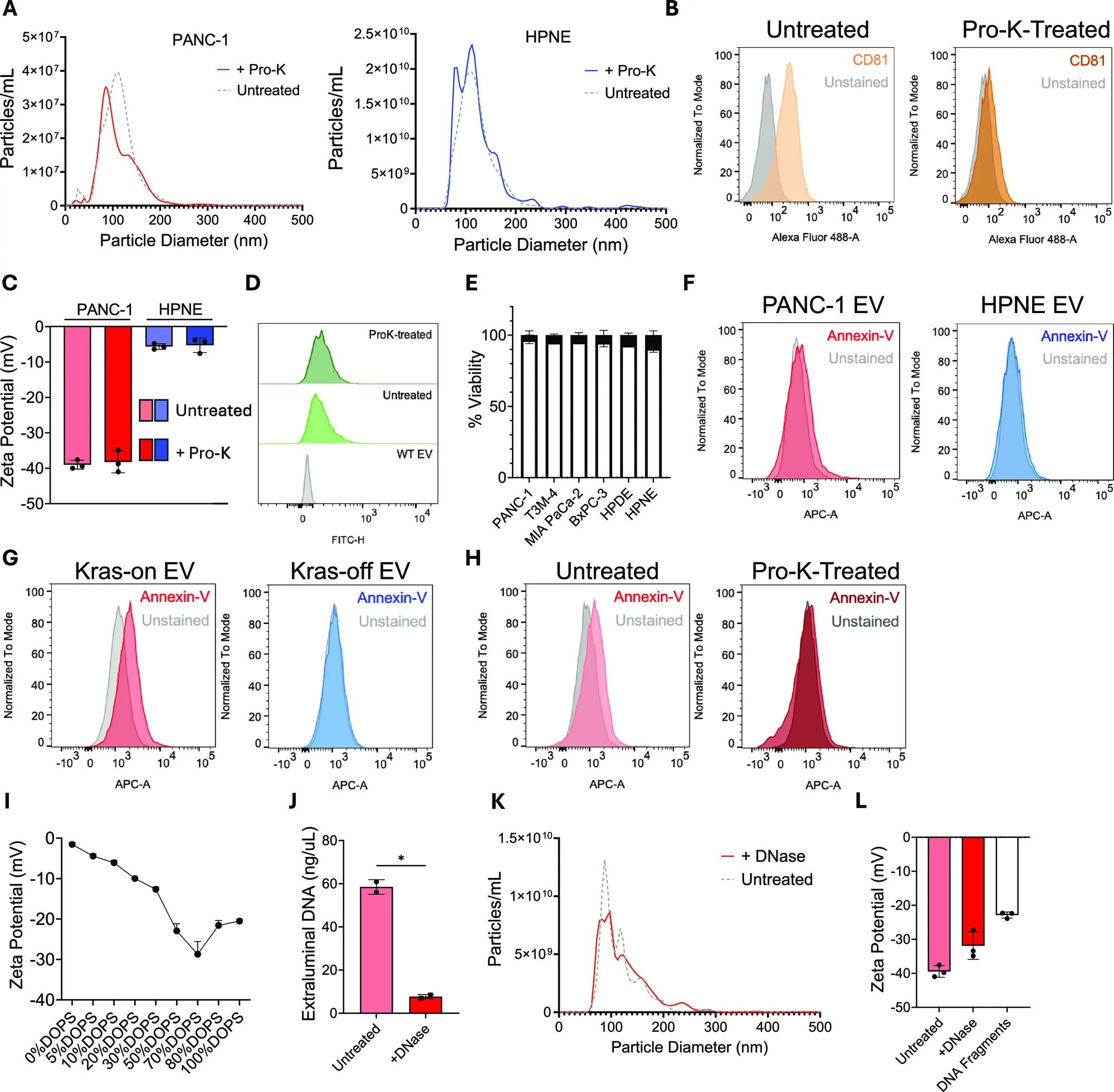
Contribution of distinct biomolecules to EV charge. (A) Particle size and concentration before and after Proteinase-K (Pro-K) treatment on EVs derived from tumorigenic (PANC-1) and nontumorigenic (HPNE) cells. (B) Efficiency of proteolysis as determined by measurement of surface CD81 on PANC-1 EVs by flow cytometry. (C) ζ-Potential of the untreated and Pro-K-treated EVs. (D) Detection of luminal EV cargo (GFP) with or without Pro-K treatment on PANC-1 CD63-GFP + EVs, as measured by flow cytometry. (E) Viability of serum-starved cells assessed by trypan blue assay. (F) Detection of surface PS on PANC-1 and HPNE EVs by flow cytometry, indicated by annexin V staining. (G) Detection of surface PS on EVs from Kras-on and Kras-off cell lines, by flow cytometry, indicated by annexin V staining. (H) Detection of surface PS on EVs before and after Pro-K treatment, by flow cytometry, indicated by annexin V staining. (I) ζ-Potential of liposomes with varying percent compositions of PS. (J) Quantification of cell-free/extraluminal DNA before and after DNase treatment of PANC-1 EVs. (K) Particle size and concentration before and after the DNase treatment. (L) ζ-Potential of untreated and DNase-treated EVs. Data are represented as mean ± SD. Statistical significance was determined using unpaired *t*-test (*p ≤ 0.05, **p ≤ 0.01, ***p ≤ 0.001, ****p ≤ 0.0001), *n* = 3 biological replicates.

To evaluate whether phospholipid PS contributes to the negative EV charge, we used flow cytometry to evaluate the PS levels on the EV surface by measuring the PS-binding protein annexin V (Figure 2E–I). Since PS exposure is associated with cell death, we controlled for this potential confounding factor using a Trypan Blue assay to assess cell viability. We found low levels of cell death in the cell lines used for EV production under this setting (Figure 2E). The PS positivity of tumorigenic PANC-1 cell line-derived EVs was higher compared to that of nontumorigenic HPNE cell line-derived EVs (Figure 2F). Taken together, these results point to higher PS exposure on the surface of EVs secreted from tumorigenic pancreatic cells than on EVs secreted from nontumorigenic cells.

Earlier, we demonstrated that EVs derived from the mutant iKras cell line (oncogenic Kras-on) possessed an overall more negative ζ-potential compared to EVs derived from oncogenic Kras-off cells. Therefore, we next evaluated whether the PS level on these EVs varied depending on their oncogenic Kras status. We observed that the EVs derived from oncogenic Kras-on cells had a higher amount of annexin V staining than those from oncogenic Kras-off cells, indicating that activated oncogenic Kras is associated with the presence of PS on the surface of cancer EVs (Figure 2G).

To explore potential reasons EVs maintain their anionic ζ-potential after Pro-K treatment, we further examined the PS level on EVs and observed that the PS was not affected by Pro-K treatment (Figure 2H). The continued anionic ζ-potential of EVs treated with Pro-K was likely partially due to the continued presence of PS. To evaluate whether anionic phospholipids apart from PS could potentially contribute to the overall negative ζ-potential of EVs, we synthetically fabricated liposomes with increasing percentages of PS and determined the ζ-potential of such liposomes. Size-distribution analysis confirmed that liposome formulations used for ζ-potential measurements were within a comparable nanoscale range (Figure S1A). We found that synthetically fabricated liposomes with 70% PS obtained the largest negative ζ-potential, but still below that of tumorigenic cell line-derived EVs (Figure 2I). These findings indicate that PS contributes to EV anionic charge, but that the relationship between PS abundance and measured ζ-potential is nonlinear, supporting the rationale that membrane organization and interfacial ion shielding influence the effective surface charge of lipid vesicles. Overall, these results indicate that PS is not affected by Pro-K treatment and the negative ζ-potential of Pro-K-treated EVs may be due to the continued presence of PS. The results with fabricated liposomes suggest that other anionic lipids or DNA on the EV membrane could additionally contribute to the overall negative ζ-potential of cancer EVs.

To investigate whether extraluminal DNA on EVs may contribute to the observed negative ζ-potential, the EV surface charge was compared before and after DNase treatment (Figure 2J–L). DNase treatment successfully removed the extraluminal DNA adhering to the EV surface (Figure 2J). We observed that DNase treatment did not alter the size or the number of EVs (Figure 2K). The smoothing of NTA peaks following enzymatic treatment may also reflect partial removal of aggregated EVs that can overlap with nonaggregated EVs in this size range. Interestingly, we observed a trend toward higher ζ-potential of the DNase-treated EVs (Figure 2L). This result indicates that extraluminal DNA may contribute to the overall negative charge on the EV surface.

### On-Chip MEP Platform Enriches Anionic EVs

We measured the ζ-potential of EVs extracted from the serum of PDAC patients and healthy donors and found no significant difference between their ζ-potential (Figure 3A). Since a highly negative ζ-potential is a distinguishing property of PDAC cell line-derived EVs (Figure 1A,I), we hypothesized that cancer EVs may constitute a much smaller fraction of total serum EVs. Serum-derived EVs in cancer patients are a heterogeneous mix of cancer and normal tissue-derived EVs, varying by patient. To assess whether cancer and noncancer EVs in patient serum exhibit distinct ζ-potentials, as seen in cell lines, we needed a method to enrich EVs based on their charge, as standard isolation techniques do not enable charge-based separation. To achieve this goal, we developed a MEP platform to selectively enrich for EVs with more negative ζ-potential values (Figure 3B).

**Figure 3:**
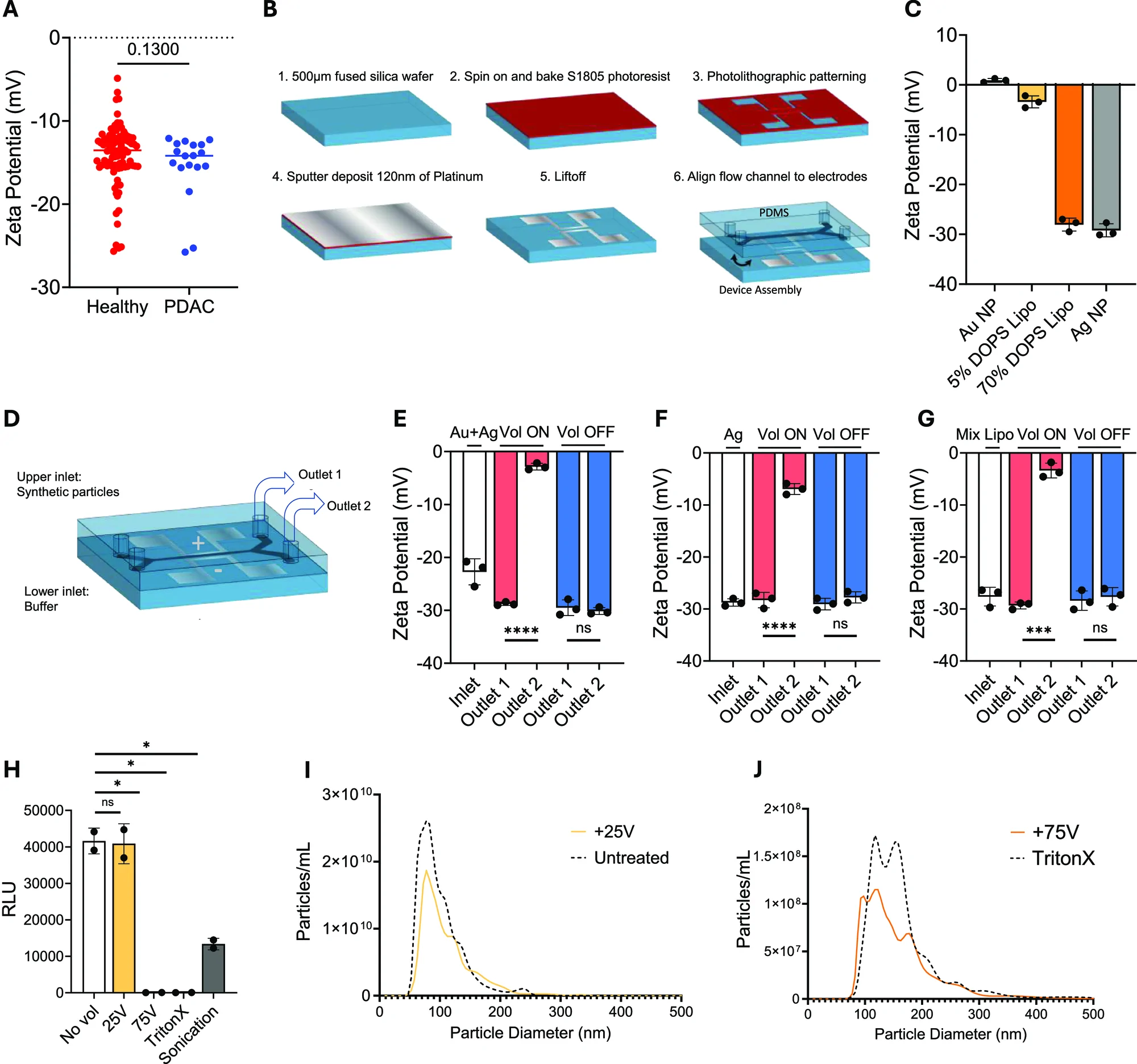
An on-chip MEP platform designed to enrich anionic nanoparticles. (A) ζ-Potential of serum-derived EVs from healthy individuals and PDAC patients (*n* = 3 technical replicates). (B) Schematic of the fabrication process of the MEP chip. (C) ζ-Potential of silver (Ag) nanoparticles, gold (Au) nanoparticles, and anionic liposomes with 5% or 70% DOPS. (D) Operational MEP diagram, where nanoparticles and buffers are loaded via the upper inlet and lower inlet, respectively, and an electric field is applied across the microfluidic channel. (E–G) ζ-Potential showing selective enrichment of mixed Ag and Au nanoparticles, enrichment of anionic Ag nanoparticles, and enrichment of highly anionic liposomes (70% DOPS + 30% DOPC), respectively. (H) Luminescence intensity of EVs with membrane-anchored luciferase after specific treatment. (I,J) Particle size and concentration of EVs after specific treatment. Data are represented as mean ± SD. Statistical significance was determined using unpaired *t*-test (*p ≤ 0.05, **p ≤ 0.01, ***p ≤ 0.001, ****p ≤ 0.0001), *n* = 3 biological replicates unless specified.

We validated the effectiveness of our MEP platform to separate based on charge using synthetic NPs. The synthetic NPs employed for validation were gold (Au) NPs, silver (Ag) NPs, and liposomes (5% DOPS and 70% DOPS). Liposomes with 5% DOPS had a charge near neutral, −4.3 ± 1.4 mV, and liposomes with 70% DOPS were highly anionic at −30.1 ± 2.1 mV (Figure 3C). The suspension of NPs and buffer was loaded via upper and lower inlets, respectively, and an electric field was applied across the horizontal microfluidic channel (Figure 3D). The separation was quantitatively assessed by measuring the ζ-potential of the recovered EVs from both outlets for both voltage-on and voltage-off conditions (Figure 3E–G). In the voltage-on condition, more anionic particles were recovered from outlet 1. However, in the voltage-off condition, no significant differences in the ζ-potential of the recovered outlets were observed for the different synthetic NPs employed here. The results demonstrate an enrichment of negative particles in outlet 1 due to the electrophoretic enrichment.

### Evaluation of EV Integrity after Exposure to the MEP Electric Field

For electrophoresis-driven enrichment, the application of an electric field may damage EVs and compromise their surface-membrane integrity. In this study, the electric field EVs experience is approximately 330 V/cm, which yields a transmembrane potential (*V*_tm_) of 2.5 mV for an average 100 nm EV. This transmembrane potential is lower than the potential required to induce electroporation or cause cellular lysis. While the applied voltage in the MEP platform did not appear to affect the EVs, as an additional validation, we tested the integrity of EVs after application of the electric field by employing EVs that were tagged with membrane-anchored luciferase with their active domains exposed on the surface. EVs were subjected to a 25 V pulse and a 75 V pulse, and the luminescence intensity was measured (Figure 3H). As a control, sonicated or lysed (Triton X treatment) EVs were used to simulate membrane rupture. In the case of the positive control, overall luminescence decreased after rupture, lysis, or under a 75 V pulse but remained unchanged in the 25 V pulse applied or untreated groups, which signifies that the membrane integrity of EVs was unaffected (Figure 3H). Size distribution determined by NTA before and after application of the 25 V pulse remained unchanged (Figure 3I), indicating the integrity of the EV surface membrane along with the unaltered luminescence intensity; however, under the 75 V pulse or TritonX treatment condition, variation in the size distribution and decrease of particle number were observed along with a decrease in luminescence intensity, which is suggestive of EV membrane damage (Figure 3I,J). The experiments here also suggest that the voltages that could induce membrane rupture as employed during electroporation are much higher than those used in our MEP devices. Overall, these results indicate that the integrity of EVs is not compromised by the voltage applied for EV enrichment by using the MEP platform.

### Enrichment of Cancer EVs Using the MEP Platform

After validating the platform with synthetic NPs, we examined the ability of the MEP device to enrich EVs with specific ζ-potential features. PANC-1 EVs, which exhibited the highest negative ζ-potential (Figure 1A), were loaded via the upper inlet at a rate of 5.5 μL/min, and buffer (EV diluent only) was loaded through the lower inlet at a rate of 11 μL/min. To confirm that large negative ζ-potential EVs migrate preferentially toward a positive electrode region, a voltage of 25 V was applied. EV concentration at both the inlet and the recovered outlets for the voltage-on/off condition was measured to evaluate enrichment of anionic EVs (Figure 4A). The concentration of EVs recovered from outlet 2 in the voltage-on condition was in the lower range of 10^7^ EVs/mL, whereas the concentration recovered from outlet 1 was in a range of 10^10^ EVs/mL, a significantly higher concentration than that of outlet 2. The NTA profile of outlet 1 recovered EVs was more representative of the small EVs when compared with outlet 2 recovered EVs in the voltage-on condition. In the voltage-off condition, the inlet EV concentration was in the range of 2 × 10^11^ to 3 × 10^11^ EVs/mL, while the particles recovered from outlets 1 and 2 had concentrations ranging between 8 × 10^10^ to 1 × 10^11^ (Figure 4B). These results suggest that the MEP platform in the voltage-on condition successfully enriched for cancer EVs and recovered them from outlet 1.

**Figure 4:**
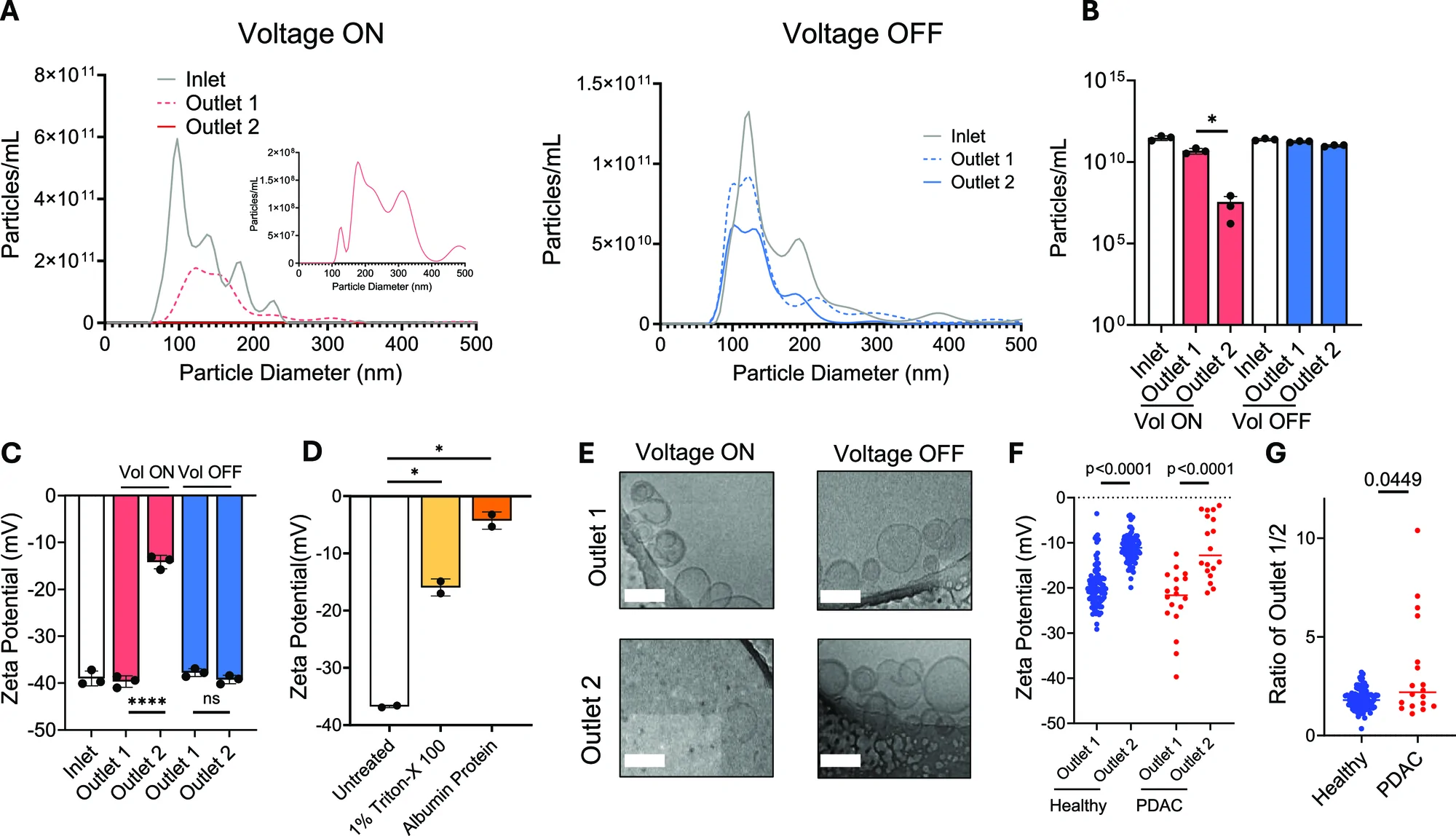
MEP platform enables enrichment of anionic EVs and discriminates PDAC patients from healthy donors. (A) Particle size and concentration for the inlet, outlet 1, and outlet 2 of the MEP device during voltage-on and -off conditions for PANC-1 EVs. (B) Quantitative characterization of EV enrichment, as shown via particle concentration at the inlet, outlet 1, and outlet 2 for voltage-on and -off conditions for PANC-1 EVs. (C) ζ-potential of particles from the inlet and outlets 1 and 2 in voltage-on and -off conditions for PANC-1 EVs. (D) ζ-potential of untreated EVs, Triton-X-treated EVs, and albumin. (E) Cryo-EM images of PANC-1 EVs showing integrity from recovered outlets, postpassage through the device in voltage on and off conditions. Scale bars, 100 nm. (F) ζ-Potential of particles recovered from outlets 1 and 2 for voltage-on conditions from serum-derived EVs of healthy individuals or PDAC patients (*n* = 3 technical replicates). (G) Ratio of ζ-potential of particles recovered from outlets 1 and 2 in voltage-on conditions from serum-derived EVs of healthy individuals or PDAC patients (*n* = 3 technical replicates). Data are represented as mean ± SD. Statistical significance was determined using unpaired *t*-test (*p ≤ 0.05, **p ≤ 0.01, ***p ≤ 0.001, ****p ≤ 0.0001), *n* = 3 biological replicates unless specified.

Next, we determined the ζ-potential of the recovered EVs from the outlets. In the voltage-off condition, no significant difference between outlets 1 and 2 was detected (Figure 4C). However, in the voltage-on condition, there was a significant difference in ζ-potential of the EVs recovered from outlet 2 compared to EVs recovered from outlet 1, suggesting the enrichment of higher anionic EVs in outlet 1 (Figure 4C). The negative ζ-potential observed in outlet 2 recovered EVs in the voltage-on condition may be attributed to the association of surface proteins and biomolecules with ζ-potential closer to net neutral values. These potential contaminants may precipitate along with EVs in the pellet when isolating EVs with the UC isolation technique. EVs generally acquire their negative surface charge from anionic phospholipids in the membrane, whereas proteins and other biomolecules acquire their electric charge from ionizable amino acid side chains and functional groups. These biomolecules usually possess an overall negative ζ-potential that is lower in magnitude than EVs.^42^

Albumin (BSA) is the most abundant protein in the blood and lysed EVs. To exclude the influence of BSA on the ζ-potential, we next evaluated the ζ-potential of ultracentrifuge-isolated EVs, lysed EVs, and BSA. We found that intact cancer EVs possessed significantly more negative ζ-potential (−36.9 ± 0.3 mV) compared to membrane-ruptured cancer EVs (−15 ± 2 mV) or BSA (−5.34 ± 2.13 mV) (Figure 4D). These results indicate efficient recovery of EVs with high negative ζ-potential and exclusion of ruptured EVs and BSA in outlet 1. Cryo-EM was performed to further characterize the particles recovered from outlets 1 and 2 for voltage-on/off conditions (Figure 4E). A large number of particles ranging in size from 80 to 150 nm were detected in outlet 1 in the voltage-on condition with morphology consistent with EVs (Figure 4E). Cryo-EM did not detect any EV-like structures in the samples collected from outlet 2 in the voltage-on condition, while in the voltage-off condition, EVs were detectable in both outlets by cryo-EM (Figure 4E).

Based on the optimization using synthetic NPs and cell-line-derived cancer and normal EVs, we proceeded to test the MEP device with EVs derived from healthy and PDAC patient serum samples to determine whether the MEP platform could enrich cancer EVs from serum. Serum EVs were fractionated using the MEP platform, and the size of EVs before and after the enrichment using the MEP platform was evaluated (Figure S2A). ζ-Potential was determined for EVs recovered from the two outlets (Figure 4F,G). In the voltage-on condition, EVs recovered from both healthy donors and PDAC patients showed robust charge-based separation, with significantly lower ζ-potential particles consistently eluting in outlet 1 compared to outlet 2 (*p* < 0.0001) (Figure 4F). This confirms that the device is functioning as intended and can resolve EV subpopulations based on surface charge. However, when comparing between groups, the ζ-potential distributions for PDAC and healthy donor EVs were not significantly different within either outlet (outlet 1: *p* = 0.1568; outlet 2: *p* = 0.8743), highlighting the limitation of raw ensemble ζ-potential values for direct clinical discrimination. To address this, we performed an enrichment analysis using the ratio of ζ-potential values between outlet 1 and outlet 2 under voltage-on conditions, termed the MEP metric (Figure 4G). This within-sample metric captures the relative shift in EV charge distribution and reveals a significant enrichment of more anionic EV populations in PDAC compared to healthy controls (*p* = 0.0449), demonstrating discriminatory ability not evident in bulk measurements alone and suggesting that the MEP platform enables selective enrichment of low ζ-potential EVs from PDAC serum, positioning ζ-potential as a complementary biomarker for PDAC detection.

To further assess MEP performance, we determined the optimal MEP metric cutoff value by Youden’s J statistic as 3.318 (*J* = 0.33) and assessed the ability of the MEP metric to discriminate between sample types (Figure S2B). Notably, both pancreatitis samples (*n* = 2) were assigned as negative at the Youden-defined cutoff (2/2; 100% classified below threshold), reinforcing the ability of this biophysical metric to distinguish malignant from inflammatory states. At a lower, sensitivity-optimized cutoff (2.05), one pancreatitis sample exceeded the threshold (1/2; 50% classified above threshold), illustrating the expected trade-off between sensitivity and specificity. These findings are consistent with the potential use of the MEP metric as a biophysical, specific “rule-in” marker for PDAC, although interpretation is limited by the small pancreatitis sample size.

Receiver operating characteristic analysis of the MEP metric demonstrated modest discriminatory ability [area under the curve (AUC) = 0.65, 95% confidence interval (CI) = 0.48–0.81, *n*_healthy_ = 92, *n*_pdac_ = 18] (Figure S2C). To assess the impact of class imbalance, repeated downsampling sensitivity analysis showed a median AUC of 0.648 (range = 0.515–0.762, repetitions = 1000, *n*_healthy_ = 18, *n*_pdac_ = 18), indicating that the observed signal is not driven by the larger cohort of healthy donor samples. Additionally, bootstrap analysis of the full data set yielded a 95% CI of 0.482–0.817, reflecting modest but variable discrimination in this pilot cohort. We further report the effect size for the MEP metric (Cohen’s *d* = 0.766), indicating a moderate-to-large separation between PDAC and healthy samples relative to within-group variability.

Triplicate ζ-potential measurements demonstrated low intra-assay variability across samples (Figure S2D). For outlet 1, the mean standard deviation was 1.26 mV with a mean coefficient of variation (CV) of 7.02%, with 85% of samples (95/112) exhibiting CV < 10%. Similarly, outlet 2 measurements showed a mean standard deviation of 0.76 mV and a mean CV of 8.53%, with 79% of samples (89/112) below 10% CV. These findings demonstrate robust technical precision of the ζ-potential measurements underlying the MEP biophysical metric. Bland–Altman analysis of technical replicate ζ-potential measurements (R1 vs R2) demonstrated a mean bias of 1.04 mV with limits of agreement from −1.86 to 3.94 mV for outlet 1, and a smaller bias of 0.21 mV with tighter limits of agreement from −1.88 to 2.29 mV for outlet 2, supporting good agreement between replicate measurements (Figure S2E,F).

To assess internal generalizability, we performed 5-fold stratified cross-validation, repeated 5-fold stratified cross-validation, and leave-one-out cross-validation. A single 5-fold stratified cross-validation yielded an AUC of 0.618 ± 0.169 across the five held-out folds. Because fold-level estimates were variable due to the small number of PDAC samples per fold (*n* = 3–4), we also performed repeated 5-fold stratified cross-validation using 1000 random fold assignments, which yielded a repeat-level mean AUC of 0.650 ± 0.019. Leave-one-out cross-validation yielded an AUC of 0.650. Together, these analyses support modest but internally reproducible discriminatory performance, while highlighting the need for cautious interpretation given the limited PDAC sample size. We also acknowledge that variability in ionic strength may contribute to differences in absolute ζ-potential values and report a median conductivity of 0.762 mS/cm (IQR: 0.371–1.38 mS/cm) during ζ-potential analyses (Figure S2G). Representative ζ-potential distributions are also found in Figure S3.

As an exploratory multivariate analysis, we evaluated whether combining the MEP metric with outlet 1 ζ-potential, outlet 2 ζ-potential, and bulk predevice ζ-potential improved discrimination between healthy and PDAC samples. Using repeated 5-fold stratified cross-validation, the combined multivariate model yielded an AUC of 0.807 ± 0.025, reported as mean ± SD across 1000 repeat-level mean AUCs. This corresponded to a ΔAUC of 0.158, suggesting that integrating device-derived and ζ-potential measurements may improve discriminatory performance in this pilot cohort. Taken together, these findings support the utility of the MEP platform as a complementary enrichment tool and “rule-in” feature to improve the detection of cancer-derived EV signatures in complex biofluids. Our ultimate goal of enriching cancer EVs from a pool of PDAC serum EVs via variations in the ζ-potential between cancer EVs and noncancer EVs was achieved in this proof-of-concept study using our MEP device.

## Conclusions

The potential of using EVs and exosomes as biomarkers in cancer detection has not been fully realized because current isolation techniques cannot reliably and rapidly distinguish between cancer EVs and noncancer EVs in circulation. Existing immunoaffinity-based methods for specific EV selection require specific antibodies or labels. Markers for isolation often capture EVs and non-EV contaminants, thus producing low or undetermined EV enrichment ratios due to inherent heterogeneity. Our work addresses these key challenges associated with cancer EV enrichment. We developed a label-free EV enrichment platform and demonstrated the feasibility of enriching EVs based on charge. Here, we have shown differential electrical properties of normal and cancer cell-derived EVs, showing that PaCa cell-derived EVs possess a larger negative net charge compared to noncancer cell-derived EVs. However, because conductivity and pH were not prospectively standardized, we consider this to be a limitation.

Our findings about surface charge of EVs, derived from both cell lines and serum, support previous findings for different cancer cell-derived EVs and body fluids.^43,44^ Akagi et al.^43^ demonstrated that EVs secreted from six cancer cell lines possessed a large negative charge compared to the noncancerous counterparts.^42^ Several studies^45–53^ have shown that EVs carry an overall negative ζ-potential; therefore, we would not expect EVs to interact with negatively charged cell membranes, yet such interactions have been documented. One possible explanation for such EV–cell interaction is that cell membranes are asymmetric and have a nonuniform lipid distribution. Some regions of the membranes are rich in anionic or cationic lipids. A cationic lipid, sphingosine, has been shown to be involved in the internalization of anionic DNA through electrostatic interaction.^45,54–58^ This suggests that the negatively charged EVs are attracted to cationic microdomains on the membrane surface and may succeed in crossing the lipid membrane barrier and get internalized in the recipient cells. Emerging single-particle techniques for EV surface charge characterization will enable improved resolution of EV heterogeneity and subpopulation-specific electrostatic properties.

Various biomolecules on the EV surface may contribute to the overall negative surface charge. As an anionic biomolecule, we investigated whether extraluminal DNA affected the negative surface charge on EVs. Reports have identified extraluminal DNA on the EV surface.^59,60^ In this study, we show that the extraluminal DNA located on the EV surface contributed to the overall anionic EV ζ-potential. We also evaluated the role of PS in contributing to the negative surface charge of EVs. PS is a negatively charged phospholipid that is preferentially localized in the inner leaflet of the plasma membrane in normal cells, whereas tumor cells frequently have PS exposure on the outer leaflet of the cell membrane. Interestingly, it has been reported that EVs secreted from cancer cells have PS externalized on the outer leaflet and are suggested to be associated with cancer progression.^52,61–67^ Here, we observed that the PS levels measured on the surface of EVs secreted by tumorigenic PaCa cells were significantly higher compared to EVs secreted from nontumorigenic pancreatic cells. Moreover, higher negatively charged EVs showed increased levels of PS externalization on their membranes. Both our study and another recent study found that PS remained unaffected by Pro-K treatment, suggesting that anionic ζ-potential Pro-K-treated EVs may result from anionic lipids such as PS.^44^

The contribution of mutant KRAS in EV secretion and charge has been a focus of ongoing research.^24^ We assessed the contribution of mutant Kras^G12D^ in EV charge and secretion and found that the EVs secreted from Kras-on cells were significantly more anionic than EVs secreted from Kras-off cells. Interestingly, we observed that EVs secreted from Kras-off cells had a significantly lower PS level, resulting in reduced anionic ζ-potential. Likewise, a prior study has shown that the induction of mutant KRAS in colon cancer cells significantly increases EV secretion.^24^ Researchers have also found that mutant KRAS cell-derived EVs contain a higher number of tumor-promoting proteins, such as KRAS, SRC family kinases, integrins, and EGFR.^24^ Previous studies have shown that KRAS has a single farnesyl chain preceded by a polybasic domain of six lysine residues.^68^ This cationic polybasic domain promotes the association of KRAS with anionic phospholipids in the inner leaflet through electrostatic interactions.^58,68–70^ In our study, EVs from Kras-on cells possessed a significantly larger negative ζ-potential and higher PS level on their surface when compared to EVs derived from Kras-off cells. Furthermore, in cells, it has been reported that positively charged polybasic domains of KRAS protein near the plasma membrane attract anionic phospholipids other than PS, such as PIP2 and PIP3, resulting in net negative surface charge on the cellular membranes.^68–72^ Future studies will likely unravel systematically the molecular determinants of EV surface charge in the context of oncogenic KRAS signaling.

One of the hallmarks of the most common type of PaCa, PDAC, is the presence of large intratumoral heterogeneity,^4,73^ which makes the disease difficult to diagnose early. Current biomarkers, such as CA19-9, do not have sufficient sensitivity.^4,74–76^ EVs have the potential to play a major role in the early diagnosis of PDAC. We developed a platform to enrich cancer EVs from serum by exploiting the electrophoretic mobility difference between cancer cell-derived EVs and normal cell-derived EVs. To test this concept, we used clinical samples to validate the ability of the MEP platform to enrich cancer EVs from circulating EVs and to reliably detect variations in the ζ-potential between healthy and PDAC donor samples. Interassay variability, batch effects, and multioperator reproducibility were not assessed and will require repeated independent runs of the same samples across operators and experimental days. The search for consistent stand-alone biomolecular markers found on the EV surface has proven to be very difficult.^12,77,78^ While a significant enrichment of more negatively charged EVs is observed through shifts in the ζ-potential ratio captured by our MEP metric, we note that the bulk ζ-potential distributions of PDAC and healthy EV populations remain overlapping under the current conditions, indicating that ζ-potential alone is insufficient as a standalone diagnostic biomarker. In addition, the limited sample size, cohort imbalance, and absence of independent cross-validation may reduce statistical power and limit reproducibility and generalizability. Thus, we envision the MEP metric as a “rule-in” feature, and ζ-potential-based enrichment as an upstream fractionation strategy that enhances downstream analyses, such as proteomic profiling or emerging AI-assisted spectroscopic approaches,^79–81^ which may provide improved sensitivity and specificity for PDAC detection.

From a clinical translation perspective, the current workflow is compatible with standard blood processing pipelines. In a representative use case, ∼500 μL of patient serum is processed via centrifugation, filtration, and UC (∼3 h) to isolate EV-enriched fractions, which are then resuspended in phosphate-buffered saline (PBS), washed once more, and then introduced into the MEP device. Charge-based fractionation is completed within 30–90 min, depending on the mobility of the EVs. Downstream ζ-potential readout can be obtained within ∼10 min using a ZetaSizer, enabling a total postisolation analysis time of under 1 h. While UC remains a time-limiting step, this preprocessing is consistent with current EV workflows and could be replaced by faster isolation methods (e.g., SEC or TFF) in future implementations. Prospective studies must evaluate this platform in larger, clinically diverse cohorts, including benign pancreatic diseases (e.g., pancreatitis) and other confounding conditions, and benchmark performance against established markers.

In summary, our work provides a promising approach for the enrichment of cancer-cell-derived EVs based on anionic charge, with potential applications for early detection of PaCa.

## Methods/Experimental

### Cell Culture

Tumorigenic and nonmalignant cells used for the study are listed in Table 1. The human cell lines used in this study were: PANC-1; MIA PaCa-2; T3M-4; BxPC-3; HPDE; and HPNE. MDA-MB-231 expressing a plasma membrane-anchored Metridia luciferase (pMetLuc) reporter; CD63-GFP-labeled PANC-1 cell line-derived EVs were used for the integrity assay. PANC-1, T3M-4, BxPC-3, HPNE, and CD63 GFP-labeled PANC-1 cells were cultured in RPMI 1640 (Corning) supplemented with 10% fetal bovine serum (FBS) and 1% penicillin–streptomycin. The primary murine cells (iKras) were isolated from p48 cre; R26-rtTa-IRES- EGFP; TetO-KrasG12D p53L/L (PiKP) PaCa genetically engineered mouse model^41^ and maintained in RPMI-1640 (Corning), 10% Tet System Approved FBS (Clontech 631106), 1X Penicillin–Streptomycin (Corning 30-002-Cl) in the presence of 1 μg/mL doxycycline to ensure oncogenic Kras expression (Kras-on cells). To extinguish oncogenic Kras, doxycycline was withdrawn from the media (Kras-off cells). HPDE cells were cultured in 50% DMEM (Corning) supplemented with 10% FBS, and 1% penicillin–streptomycin and 50% defined keratinocyte-SFM (Gibco) with 1% penicillin–streptomycin. MDA-MB 231 pMetLuc membrane reporter cells (metridia membrane-bound luciferase). Coelenterazine (Fisher Scientific) was used as the substrate for the MDA MB-231 pMetLuc cells, and luminescence intensity was measured after 2 min at room temperature (RT) using a luminometer (BMG Labtech) Omega Microplate reader. All cell lines were tested for mycoplasma contamination and were maintained in humidified cell culture incubators at 37 °C and 5% CO_2_.

**Table 1:** Cell Lines

cell line	growth conditions	source	malignant
PANC-1	RPMI-10% FBS, 1% PS	ATCC	yes
MIA PaCa-2	DMEM-10% FBS, 2.5% Horse Serum, 1% PS	ATCC	yes
T3M-4	RPMI-10% FBS, 1% PS	Riken Bioresource	yes
BxPC-3	RPMI-10% FBS, 1% PS	ATCC	yes
HPDE	1:1 Keratinocyte serum-free media: DMEM, 10% FBS, 1% PS	MDACC	no
HPNE	RPMI-10% FBS, 1% PS	ATCC	no
iKras (Murine Kras^G12D^ On or Off)	RPMI 10% Tet-FBS, 1% PS	MDACC	yes
MDA MB-231 pMetLuc membrane reporter	DMEM 10% FBS, 1% PS	MDACC	yes
PANC-1 CD63-GFP+	RPMI-10% FBS, 1% PS	MDACC	yes

### Generation of pMetLuc and CD63 GFP + Cells

MDA-MB-231 cells were transfected with pMetLuc-Mem Control (Clontech) with Lipofectamine 2000 according to manufacturer’s instructions. Cells were selected with 2 mg/mL G418 (Gold Biotechnology) to establish a stably transfected line. PANC-1 (1 × 10^3^ cells) were transduced in a 96-well plate with 1 μL of pCT-CD63-GFP lentivirus (SBI CTYO12-VA-1). Cells were then selected with 5 μg/mL puromycin (Sigma-Aldrich) to establish a stable line.

### Ras Activation Assay

Ras activation was measured using a Ras Activation ELISA Assay Kit (MilliporeSigma) following the manufacturer’s instructions.

### Cell Line-Derived EV Production

Cells were grown in T225 flasks to 85% confluence and were washed two times with 1X PBS. Then, the cells were incubated for 48 h in serum-free media. The conditioned media were centrifuged for 5 min at 800*g* and then for 10 min at 2,000*g* to remove cells and smaller debris. The supernatant was filtered through a 0.2 μm pore filter (Corning, Cat. No. 431219) and then subjected to UC (Beckman) at 100,000*g* for 3 h at 4 °C in a Beckman SW 32 Ti rotor. The supernatants were discarded, and the pellets containing EVs were resuspended in 1× PBS for downstream processing.

### Patient Samples and Tissue Collections

Serum samples from healthy participants were obtained as deidentified, discarded clinical material and were therefore exempt from Institutional Review Board (IRB; PA14/0A32) approval. Informed consent was obtained from all participants in accordance with institutional guidelines. Blood samples of healthy individuals were collected by MD Anderson Blood Bank. Samples were extracted after centrifugation at 3,000*g* for 20 min and stored at −80 °C until analyzed. Serum samples from patients diagnosed with PDAC were collected at the University of Cologne and University Hospital Heidelberg by the local IRB (Cologne: 13-091; Heidelberg: 323/2004), and written informed consent was obtained from all participants prior to surgery in accordance with institutional guidelines. The information on treatments and stage of tumor is listed in Table S1. Blood samples were obtained preoperatively, prior to any surgical intervention. Samples were extracted after centrifugation at 2,500*g* or at 4000 rpm for 10 min and stored at −80 °C until analyzed.

A total of 92 healthy participants, 2 pancreatitis patients, and 18 PDAC patients were included in this study with a gender-balanced distribution.

### Human Serum-Derived EV Production

500 μL of serum sample per donor (*n* = 112 total) were rapidly thawed at 37 °C in a water bath. The serum was centrifuged at 4 °C at 500*g* for 5 min, and 2,000*g* for 10 min, and filtered in a syringe with a 0.22 μm membrane filter directly into small ultracentrifuge tubes (Beckman Coulter). PBS was added to the ultracentrifuge tube to fill up to 11 mL. Samples were ultracentrifuged in a SW 40 Ti swinging bucket rotor at 100,000*g* for 3 h at 4 °C. In the wash step for serum EVs, the EV pellet was resuspended in PBS, the ultracentrifuge tubes were then filled to the top with PBS, and samples were ultracentrifuged again in a SW 40 Ti swinging bucket rotor at 100,000*g* for 3 h at 4 °C. Supernatant was aspirated and pellets were resuspended in 500 μL PBS and stored at −80 °C until further use.

### NP Tracking Analysis

NP size and concentration were measured by NTA using NanoSight instruments (Malvern Panalytical). EV samples were analyzed on an LM10 or NS300 system equipped with a 488 nm laser and sCMOS camera. Samples were diluted in cell culture grade water to achieve optimal particle concentrations for analysis. Measurements were performed at 25 °C using a camera level of 14–15, with a constant detection threshold applied across all samples. Syringe pump speeds were set to 20 for EVs and 80 for liposomes. For each sample, three 30–90 s videos were recorded and analyzed, and the mean values were used to determine particle size distribution and concentration.

### ζ-Potential Measurement

ζ-Potential of EVs was determined with a Malvern Zen 3600 Zetasizer. Anionic and cationic liposomes were fabricated as controls, and 5 × 10^9^ particles/mL were tested to generate a standard curve followed by measurements for EVs. A folded capillary zeta cell (DTS 1070) from Malvern Paranalytical was used. All EVs were diluted in CCGW to a concentration of 5 × 10^9^ particles/mL for cell culture samples or 6.25 × 10^10^ particles/mL for serum samples. Three biological and/or three technical replicates were performed, with settings (dielectric constant = 78.5; viscosity = 0.8872; RI (refractive index) = 1.330; temperature = 25 °C).

### Cryo-EM Analysis

In order to verify the morphology of the EVs and evaluate the integrity postpassage through the chip, we employed Cryo-EM. For cryo-EM, EVs were resuspended in 50 μL of PBS. The vitrification steps were performed at the SEA (Shared Equipment Authority) of Rice University. Quantifoil mesh grids were used for the study. Before the vitrification step, the grids were glow-discharged in air for 120 s. For the vitrification process, about 3–5 μL of EV samples were applied to the grid. After blotting, the grids were plunge-frozen into liquid ethane using the FEI Vitrobot system. The frozen grids were then transferred to liquid nitrogen until imaging. Imaging was performed using a JEOL 2010 Transmission electron microscope equipped for cryogenic imaging.

### Bead-Based Flow Cytometry Analysis of EVs

To evaluate surface protein marker expression, 5 × 10^9^ EVs were resuspended in 200 μL PBS in Eppendorf tubes. 10 μL of aldehyde/sulfate latex beads (4% w/v) (Invitrogen) was added to the solution and mixed at RT for 15 min. In order to facilitate the bead binding to EVs, 600 μL PBS was added into each vial, vortexed, and allowed to rotate at 4 °C overnight. The following day, 400 μL glycine (1 M) was added to each vial and incubated at RT for 1 h with rotation. Next, the bead-bound EVs were precipitated at 12,000 rpm at RT for 2 min. Next, as a blocking step, the EVs binding the beads were incubated for 60 min in 100 μL of 10% BSA. The suspension was then precipitated at 12,000 rpm for 2 min at RT to wash away excess BSA. The EVs attaching to the beads were then resuspended in 40 μL of 2% BSA in 1X PBS and were split equally into six tubes to stain for EV surface markers (CD81, CD63, and CD9) and for control (secondary antibody only, IgG alone, and EV alone). The bead-bound EVs were incubated with unconjugated primary antibodies: 1 μL of mouse antihuman CD63 (BD 556019, diluted 1:2.5 in 2% BSA); mouse antihuman CD81 (BD 555675, diluted 1:2.5 in 2% BSA); mouse antihuman CD9 (Sigma SAB4700092, diluted 1:5 in 2% BSA); isotype control: mouse IgG1 κ isotype control (BD 555746, diluted 1:2.5 in 2% BSA). For mouse cell line-derived EVs, the following antibodies used were: rat antimouse CD63 (BD 564221, diluted 1:2.5 in 2% BSA); biotin hamster antimouse CD81 (BD 559518, diluted 1:2.5 in 2% BSA); biotin rat antimouse CD9 (BD 558749, diluted 1:5 in 2% BSA); isotype control: purified rat IgG2a, κ isotype control (BD 553927, diluted 1:2.5 in 2% BSA) in 20 μL volume with 2% BSA, and rotated for 1 h at RT. After incubating with primary antibody for 1 h, the bead-bound EVs were precipitated at 12,000 rpm at RT for 2 min, and the supernatant was discarded. After incubation with the unconjugated primary antibodies, the bead-bound EVs were washed 3× in 200 μL of 2% BSA and precipitated at 12,000 rpm for 2 min, and the supernatant was discarded. Next, the bead-bound EVs were incubated for 1 h rotating in 20 μL of 2% BSA containing the corresponding secondary antibody (donkey antimouse, Alexa Fluor 488 (Invitrogen A21202); streptavidin, Alexa Fluor 488 (Invitrogen S11223); donkey antirat, Alexa Fluor 488 (Invitrogen A21208) 1:20 in 2% BSA. After staining with the secondary antibody, the samples were then precipitated at 12,000 rpm at RT for 2 min. Finally, the bead-bound EVs were washed 3X with 2% BSA in PBS. The EV surface markers CD9, CD63, and CD81 on bead-bound EVs were analyzed by LSR Fortessa X-20 (BD Bioscience). Quantification of positive EVs for CD81, CD63, and CD9 was performed in FlowJo Version 10.6.1.

### Anchorage-Independent Growth Assay

Soft agar was used to examine anchorage-independent growth. CytoSelect 96-Well Cell Transformation Assay (Soft Agar Colony Formation Catalog# CBA-130-T) from Cell Biolabs was utilized as per the manufacturer’s protocol. Briefly, 50 μL of the base agar matrix was dispensed into the wells of a 96-well plate. At the bottom surface of each well, once the agar was solidified, a cell suspension of 75 μL containing 10 × 10^3^ cells was layered on top of the agar. 100 μL 2× complete RPMI medium with 10% FBS and 1% penicillin–streptomycin was added to the cell suspension. On the eighth day, the colony formation was examined under a light microscope. Next, the quantitation of anchorage-independent growth was performed by fluorometric detection. Eight days post incubation, in order to solubilize the agar matrix completely, 50 μL of the agar solubilization solution was added to each well and incubated for 1 h at 37 °C. In order to ensure complete agar solubilization, each well was pipetted 5–10 times. Next, 25 μL of the 8× lysis buffer was added into each well and pipetted well to ensure a homogeneous mixture. After incubation of the plate for 15 min at RT, 10 μL of the lysed mixture was transferred to a 96-well plate that was suitable for fluorescence measurement. 90 μL of the CyQuant^©^ GR dye solution was added to each plate and incubated for 10 min at RT. When CyQuant^©^ GR dye binds to the nucleic acids, it exhibits strong fluorescence enhancement. In order to determine the fluorescence from the colonies, the plate was read in a 96-well fluorometer. The fluorescence produced by each well was proportional to the number of viable cells.

### Cleavage of Surface Proteins with Proteinase-K

EVs isolated by UC were used. For the preparation of ProteinaseK (Pro-K)-digested EVs, purified EVs were incubated with 5 mg/mL of Pro-K (dissolved in RNase-free water Sigma-Aldrich P2308) at 37 °C for 30 min. This was followed by an inactivation step where ProK-treated EVs were heated at 50 °C for 20 min. EVs were washed post Pro-K treatment with PBS at 28,000 rpm for 3 h using UC in a SW40 Ti swinging bucket rotor. The washed EVs were collected, and concentration was assessed for particle size and concentration. The collected EV samples were aliquoted to limit the freeze–thaw cycles. The samples were stored at −80 °C until used to probe for biomolecular and physicochemical properties. The cleavage of the ectodomain of EV surface proteins with Pro-K treatment was validated by flow cytometry. The aldehyde/sulfate bead flow cytometry approach described above was used. EV integrity after Pro-K treatment was determined by quantifying the release of select luminal proteins. PANC-1 cells expressing intraluminal GFP were also used, and integrity was determined by comparing the GFP levels of EVs before and after treatment by a PMT detector on the LSR Fortessa X-20 flow cytometer.

### DNase Treatment of EVs

EVs were treated with 2 U/μL Turbo DNase (AM2238) in 1× TURBO DNase buffer and incubated for 30 min at 37 °C. Turbo DNase was inactivated by incubation in 2 mM EDTA and heated at 65 °C for 10 min. After DNase digestion, samples were washed in PBS and precipitated by UC at 28,000 rpm for 3 h in a SW40 Ti swinging bucket rotor. All washed EVs were collected, and concentration was assessed for particle size and concentration. Removal of extraluminal DNA on the EV surface was evaluated by measuring the DNA concentrations before and after the TURBO DNase treatment using a Nanodrop spectrophotometer.

### Lysis of EVs

Isolated EVs were lysed with 1% Triton X-100 for 30 min at 37 °C with a 5× protease inhibitor. The samples after treatment with 1% Triton X-100 were washed with PBS and spun down by UC in a SW40 Ti swinging bucket rotor at 28,000 rpm for 3 h at 4 °C. Protein quantification of lysed and intact EVs was determined by microBCA assay (Thermo Fisher Cat no. 23235).

### Assessment of PS Levels on EVs by Flow Cytometry

Similar to the EV bead-flow cytometry protocol mentioned above, briefly, 2 × 10^10^ EVs were resuspended in 200 μL of PBS, and 10 μL of aldehyde/sulfate beads (Invitrogen A37304) was added to the EVs, followed by rotation for 15 min at RT. 600 μL of PBS was added to the solution and rotated overnight at 4 °C. 400 μL of glycine was added and rotated for 1 h at RT. This mixture was precipitated at 12000 rpm at RT for 2 min. The pellet was resuspended in 100 μL of 10% BSA and mixed for 1 h. The mixture was centrifuged at 12,000 rpm for 2 min at RT, and the supernatant was discarded. The bead-bound EVs were resuspended in 40 μL of 2% BSA in PBS and split equally into two tubes: for staining with APC annexin V and for control (EV alone unstained). EVs were stained with APC annexin V (Cat no. 640941, BioLegend, 1:20) diluted in 1× nnexin V binding buffer rich in calcium ions at RT for 1 h in the dark. The bead-bound EVs were washed 3 times with 1× annexin binding buffer in PBS and analyzed for annexin V using the BD Biosciences LSR Fortessa X-20 flow cytometer. As a control, beads were also stained with APC annexin V diluted in 1× Annexin V. Analysis of positive EVs for Annexin V was performed in FlowJo Version 10.6.1, and overtone levels were determined.

### RT-qPCR for the iKras Cell Lines

For cell culture, Trizol Reagent (Invitrogen #15596026) was directly added to the cell culture plate, and total RNA was extracted using Direct-zol RNA MiniPrep Kit (Zymo Research #R2052). 2 μg of RNA was used for cDNA synthesis by a high-capacity cDNA reverse transcription kit with RNase inhibitor (Applied Biosystems #4374966). qPCR was run with Fast SYBR Green Master Mix (Applied Biosystems #4385612) and the primer sets described in Table 2 on the QuantStudio 7 Flex Real-Time PCR System. Transcripts were normalized to 18S levels. The relative fold change expression was used to express the results.

**Table 2:** Primer Sets for RT-qPCR

gene	forward primer (5′–3′)	reverse primer (5′–3′)
Kras^G12D^	ACTTGTGGTGGTTGGAGCAGC	TAGGGTCATACTCATCCACAA
18S	GTAACCCGTTGAACCCCATT	CCATCCAATCGGTAGTAGCG

### MEP Device Fabrication

Flow channel and electrode channel photomasks were designed in K-Layout Version 0.25.9 and printed by Output City CAD services. For fabricating the electrode wafer, a 500 μm-thick fused silica wafer was plasma cleaned for 30 s. S-1805 photoresist was spin-coated and soft-baked at 110 °C for 1 min. For photolithographic patterning, exposure of 95 mJ/cm^2^ was used to pattern the electrode, followed by soaking in MF 321 for 120 s with gentle agitation. The wafer was rinsed in DI water and dehydrated for 5 min at 95 °C, followed by plasma cleaning for 30 s. A 120 nm-thick layer of platinum was sputter-deposited with parameters: Pt, 25 W, 3 mTorr, Ar, 35 sccm for 60 min. After sputter deposition, an acetone lift-off for 1 h was performed on the shaker. The wafer was rinsed with DI water and dried under a stream of N_2_. The electrode wafer was then stored in a dry RT environment. For fabricating the flow channel, SU-8 3025 was spin-coated on the wafer to achieve a target height of 20 μm, followed by a prebake for 14 min at 95 °C. The flow channel pattern was exposed at 355 mJ/cm^2^ and postbaked at 65 °C for 5 min, followed by ramping at 10 C/min for 10 min at 95 °C. The wafer was developed in SU-8 developer for 8.5 min, rinsed with IPA, and dried using N_2_ gas. Finally, the flow channel wafer was hard-baked at 65 °C for 5 min, followed by ramping at 10 C/min for 20 min at 150 °C. Once polydimethylsiloxane (PDMS) (Sylgard, Dow Corning; 10:1 elastomer: cross-linker weight ratio) was cast and cured on the flow channels, it was peeled off. Holes were punched to connect the tubing. The flow channels and the electrode wafer were aligned, pressed down, and thermally bonded at 80 °C for 16 h. The completed device was then soldered to wires, and the solder connections were insulated using JB-weld epoxy.

### Device Preparation

Prior to flow, devices were functionalized overnight with 10% BSA to avoid nonspecific adsorption of EVs due to the hydrophobic nature of PDMS. An ultrasensitive quartz crystal microbalance (QCM-D, Q-Sense E4 System, Biolin Scientific) was also utilized to confirm no interaction of EVs with the BSA layer. The QCM-D sensor surface was coated with BSA, and EVs were channeled through the surface, but no significant change in frequency (mass adsorption) was observed.

For the voltage-on experiments, voltage was applied across the electrodes at 25 V by a DC power supply (Mastech HY3005D), corresponding to an electric field strength of ∼300 V/cm across a 4 mm channel length, with an estimated transmembrane voltage of ∼2.475 mV. The electrode length was 3 mm and aligned along the channel. Inlet 1 was the exosomal sample inlet, and the EV solution was passaged at a rate of 5.5 μL/min, while inlet 2 was the buffer inlet, and PBS was passaged at a rate of 11 μL/min (New Era Pump Systems NE-300). All MEP experiments were performed by a single operator. No independent cross-validation was performed.

### Synthetic NPs and Liposome Experiments

1,2-Dioleoyl-*sn*-glycero-3-phospho-l-serine [18:1 PS (DOPS)], 1,2-dioleoyl-*sn*-glycero-3-phosphocholine [18:1 (Δ9-Cis) PC (DOPC)], and 1,2-dioleoyl-3-trimethylammonium-propane [18:1 TAP (DOTAP)] from Avanti were used. Different ratios of lipids were mixed in 1 mL chloroform and then dried with N_2_ gas. The resulting lipid films were placed in a vacuum chamber for 1 h to evaporate the chloroform. Then, the lipid films were hydrated with 1 mL PBS followed by vigorous mixing. The liposomes were extruded through a 100 nm diameter polycarbonate membrane 15 times. The bulk liposome solution was stored at 4 °C for short-term storage and −80 °C for long-term storage. Thawed liposomes were assessed for particle size and concentration.

### Statistical Analysis

All data are expressed as mean values or standard deviations (SD), as indicated. Statistical analysis was performed using GraphPad Prism version 10, Excel, or Python. The statistical analysis used is specified in detail in the respective figure legends.

## Supplementary Material





## Notes

Source data for the figures given in the article (XLSX)
Supplementary EV characterization, MEP performance, and clinical data (PDF)
