# Supplementary material for: Oncogenic KRAS Regulates Secretion of Extracellular Vesicles and Surface Membrane Charge via Regulation of Phosphatidylserine in Pancreatic Cancer
Source: ACS Nano. 2026 Sep 1;20(36):24605–20. doi: 10.1021/acsnano.6c08263 (PMC13585180; doi:10.1021/acsnano.6c08263)
Supplement: Supplementary file 1 [file nn6c08263_si_002.pdf]

Supporting Information

Oncogenic KRAS regulates secretion of extracellular vesicles and surface membrane charge via regulation of phosphatidylserine in pancreatic cancer

Paul A Spezza[1,2#], Kshipra S Kapoor[1,3#], Beatrice P Pforr[1,4], Xin Luo[1], Kaira A Church[1], Martin M Bell[3,5], Bo Fan[3], Seoyun Kong[1], Elena V Ramirez[1], Yi-Lin Chen[6], Fernanda G Kugeratski[1], Sibani Lisa Biswal[6], Kathleen M McAndrews[1], Florian Gebauer[7], Christoph Kahlert[8], Jacob T Robinson[2,3,5,9], Raghu Kalluri[1,2,3,10,*]

[1] - Department of Cancer Biology, The University of Texas MD Anderson Cancer Center, Houston, Texas 77030, United States
[2] - Department of Bioengineering, Rice University, Houston, Texas 77005, United States
[3] - Department of Electrical and Computer Engineering, Rice University, Houston, Texas 77005, United States
[4] - Department of Systems, Synthetic, and Physical Biology, Rice University, Houston, Texas 77005, United States
[5] - Applied Physics Program, Rice University, Houston, Texas 77005, United States
[6] - Department of Chemical and Biomolecular Engineering, Rice University, Houston, Texas 77005, United States
[7] - Department of Surgery II, University of Witten/Herdecke, Helios University Hospital Wuppertal, 42283 Wuppertal, Germany
[8] - Department of General, Visceral, and Transplant Surgery, Heidelberg University, Heidelberg University Hospital, 69120 Heidelberg, Germany
[9] - Department of Neuroscience, Baylor College of Medicine, Houston, Texas 77030, United States
[10] - Department of Pathology, The University of Texas Medical Branch, Galveston, Texas 77555, United States

[#] - Equal contribution

[*] - **Author to whom correspondence should be addressed:** RKalluri@mdanderson.org

Supplementary Figure 1: Size and morphological characterization of EVs and liposomes.

(A) Particle concentration and size of EVs and liposomes. **(B)** Morphology of tumorigenic and nontumorigenic EVs, determined by Cryo-EM imaging. Scale bars, 100 nm. **(C)** Flow cytometry analysis of EVs for common tetraspanin EV markers with representative histograms for indicated cell lines. **(D)** Aggressiveness of tumorigenic and nontumorigenic cells tested in soft agar assay. Scale bars, 140 μm. Data are represented as Mean ± SD.

Supplementary Figure 2: Characterization, performance, and reproducibility of the MEP metric.

(A) Mode size of EVs from donors recovered from inlets and outlets under voltage on condition. **(B)** Ratio of ζ-potential of particles recovered from outlets 1 and 2 in voltage on conditions with Youden-defined and sensitivity-optimized detection cutoffs. **(C)** Receiver operating characteristic (ROC) curve for the MEP metric showing modest discriminatory ability. **(D)** Histogram of coefficient of variation (CVs) from replicate ζ-potential measurements from outlets 1 and 2 show low intra-assay variability. **(E-F)** Bland-Altman plots of replicate ζ-potential measurements (R1 vs R2) demonstrate low bias with narrow limits of agreement. **(G)** Violin plot of conductivity measurements during ζ-potential measurements.

Supplementary Figure 3: Representative ζ-potential distributions of serum EVs.

Representative ζ-potential distributions from serum EVs after passage through MEP device. n = 3 technical replicates.

Supplementary Table 1:Tumor stage and treatment history of patients with PDAC or pancreatitis.

Supplementary Figure 1

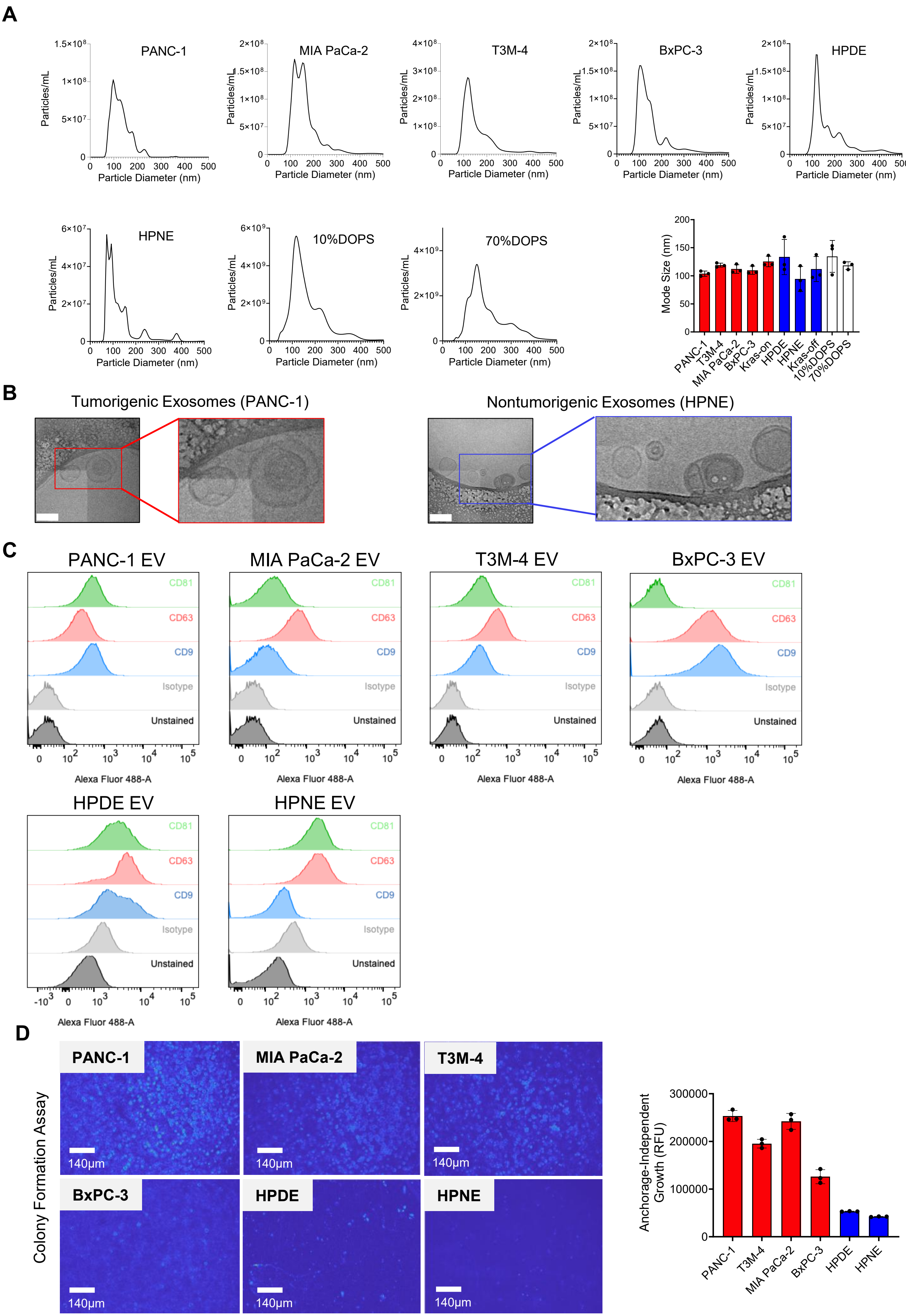

Supplementary Figure 2

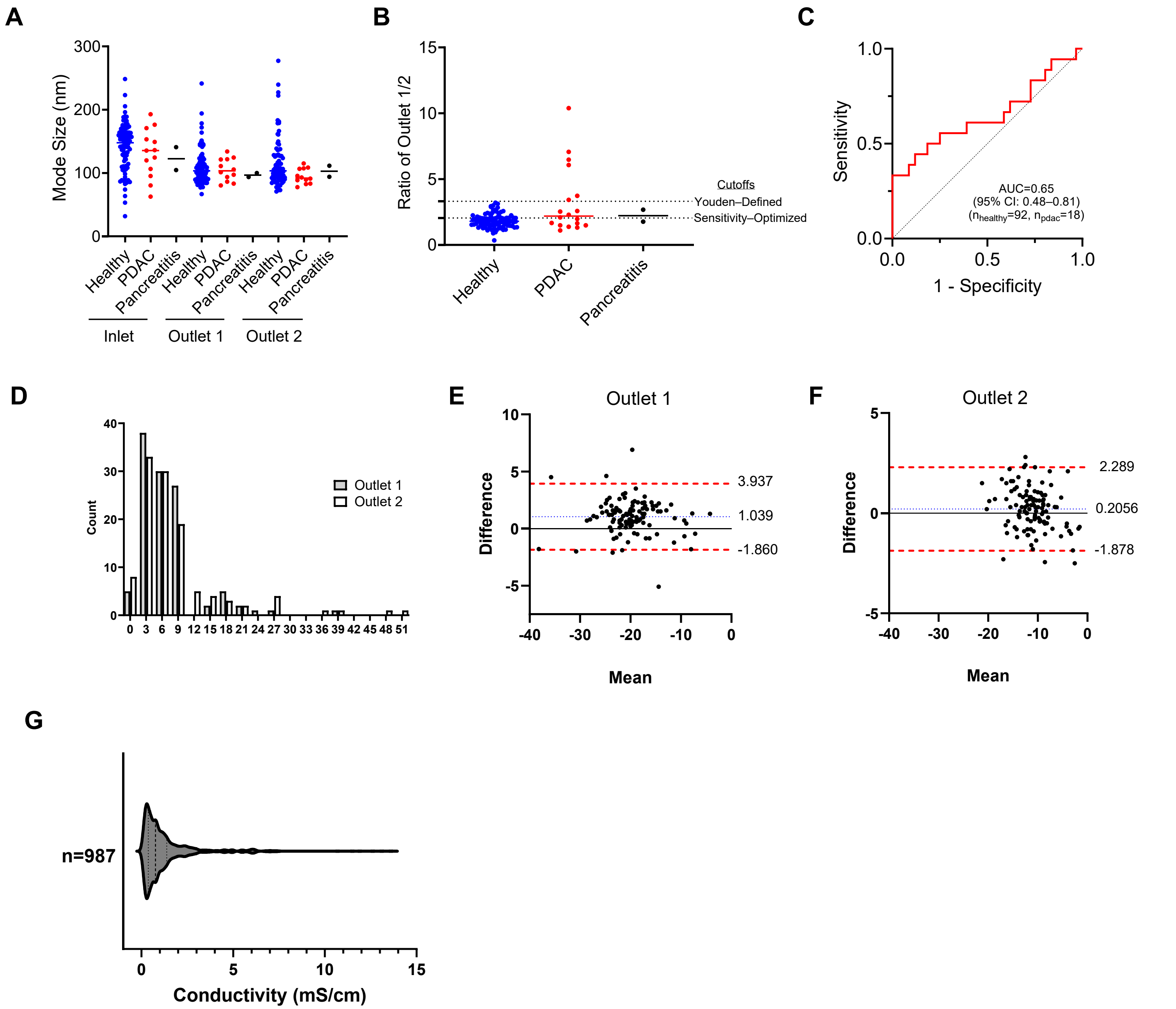

Supplementary Figure 3

Average = **-39.70 mV**

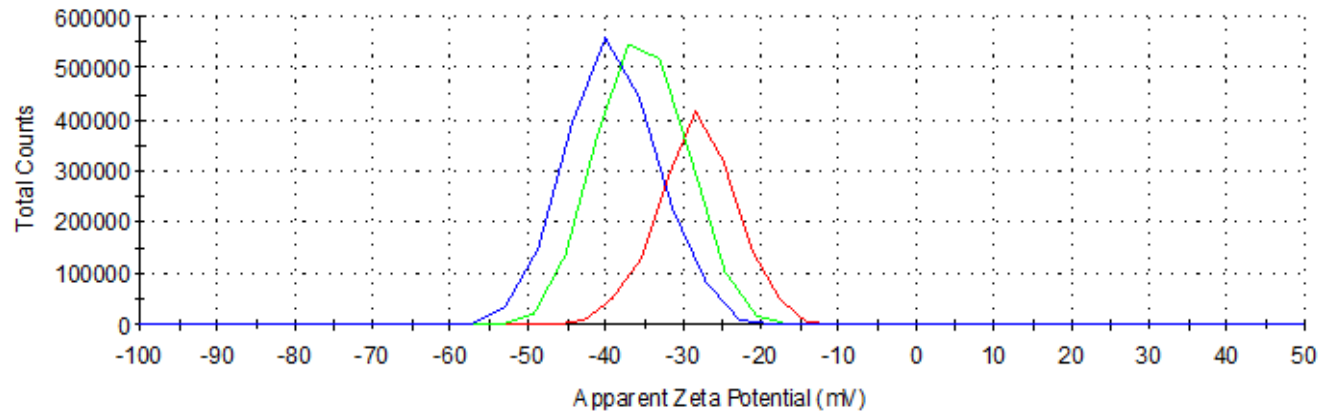

Average = **-34.57 mV**

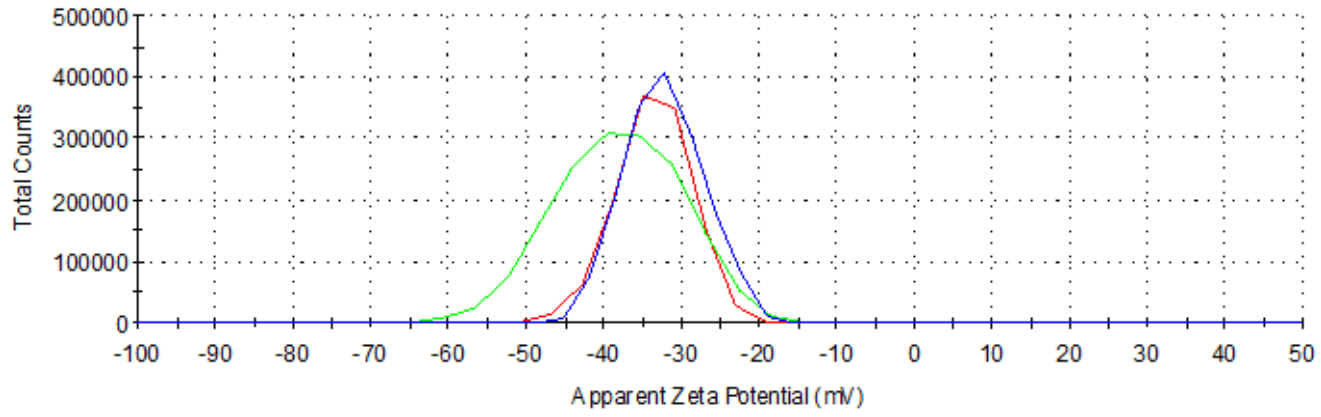

Average = **-31.97 mV**

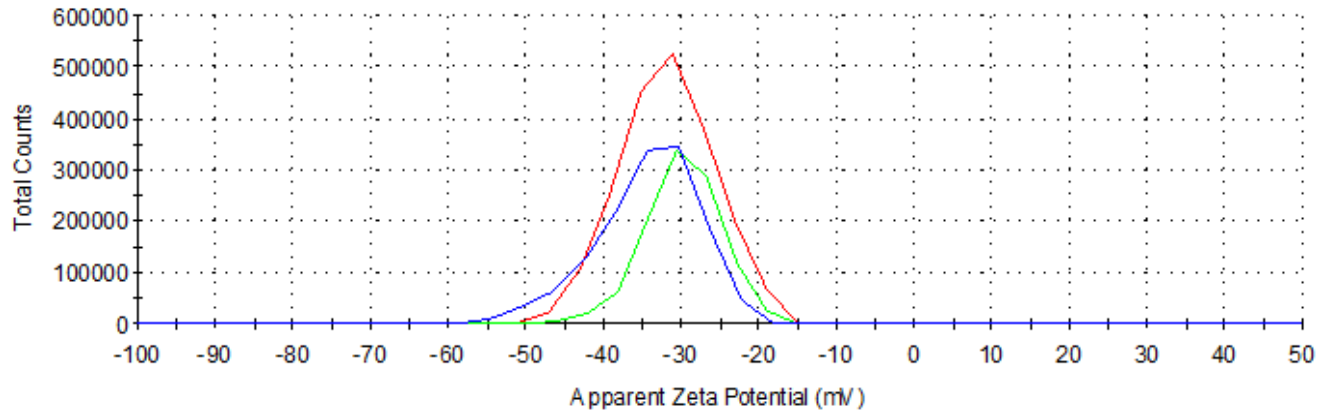

Average = **-24.83 mV**

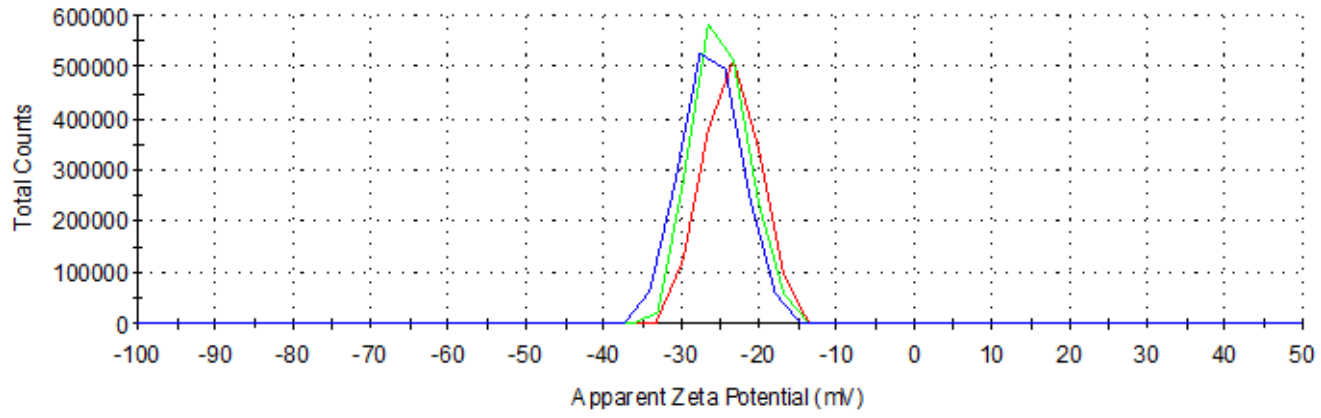

Average = **-20.37 mV**

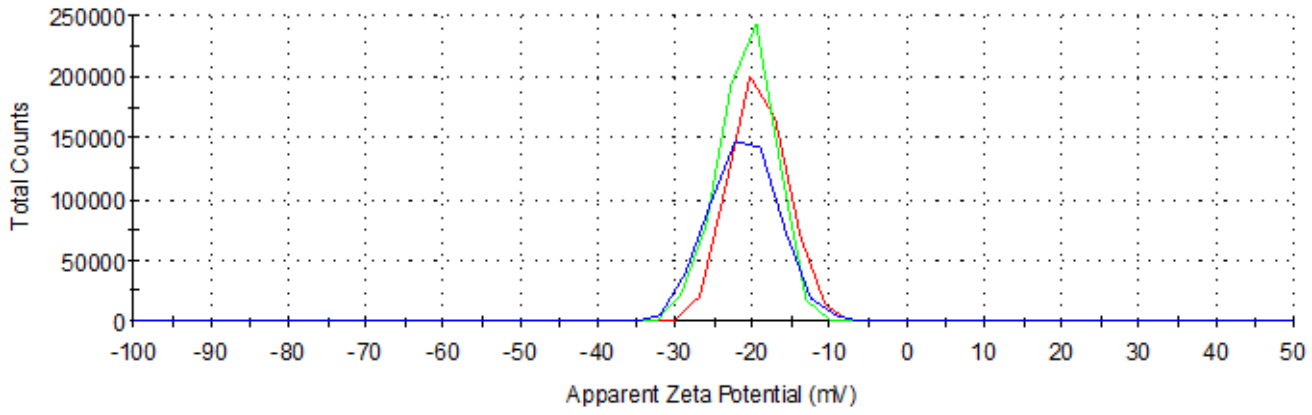

Average = **-14.87 mV**

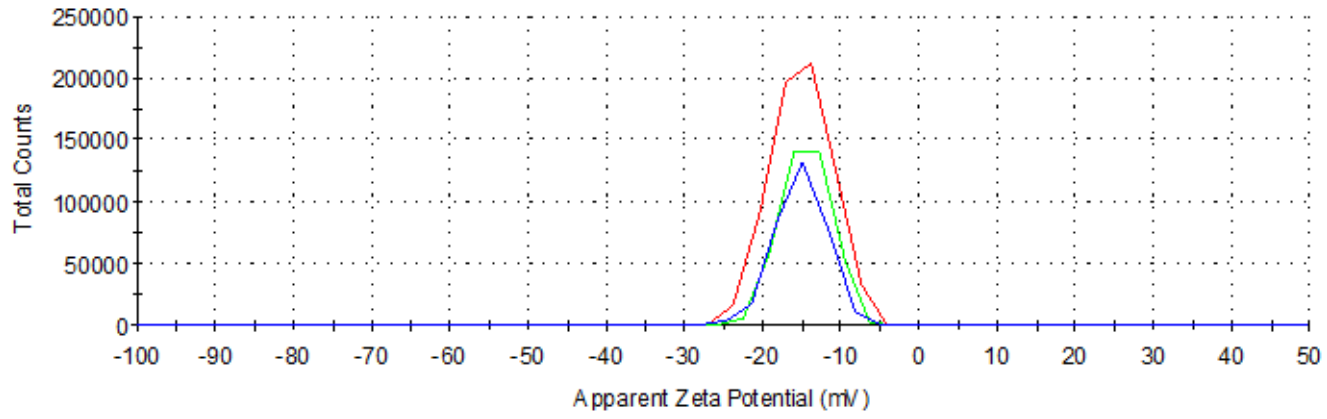

Average = **-11.83 mV**

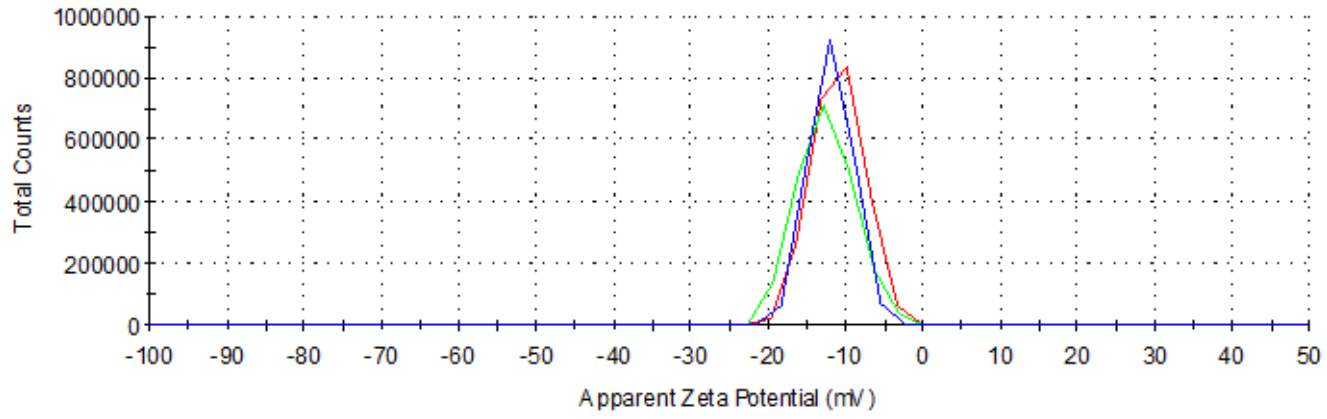

Average = **-7.46 mV**

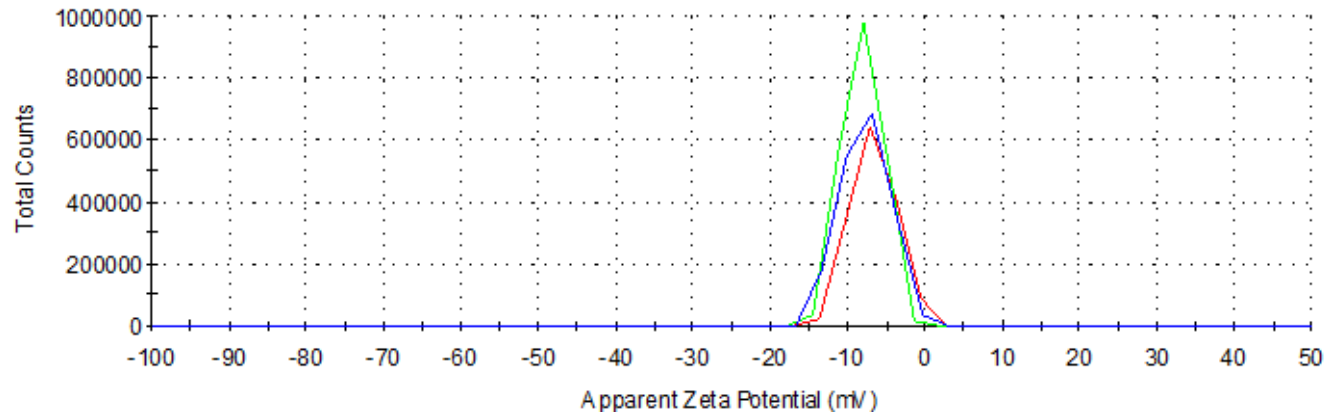

Supplementary Table 1

ID	Disease	Tumor Stage	Treatment Status	Sample Type	Notes
PG1	PDAC	II	Treatment-naïve	Peripheral Blood	
PG2	PDAC	III	Treatment-naïve	Peripheral Blood	
PG3	PDAC	III	Treatment-naïve	Peripheral Blood	
PG4	PDAC	II	Treatment-naïve	Peripheral Blood	
PG5	PDAC	III	Treatment-naïve	Peripheral Blood	
PG6	PDAC	III	Treatment-naïve	Peripheral Blood	
PG8	PDAC	III	Treatment-naïve	Peripheral Blood	
HD1795	PDAC	III	Treatment-naïve	Peripheral Blood	
HD1852	PDAC	II	Treatment-naïve	Peripheral Blood	
HD1856	PDAC	III	Treatment-naïve	Peripheral Blood	
HD1857	PDAC	III	Treatment-naïve	Peripheral Blood	
HD1887	PDAC	III	Treatment-naïve	Peripheral Blood	
HD1899	PDAC	N/A	Treatment-naïve	Peripheral Blood	Peritoneal Carcinomatosis
HD1909	PDAC	II	Treatment-naïve	Peripheral Blood	
HD1910	PDAC	II	Treatment-naïve	Peripheral Blood	
HD1912	PDAC	IV	Treatment-naïve	Peripheral Blood	
HD1919	PDAC	N/A	Neoadjuvant adio-chemotherapy	Peripheral Blood	
HD1923	PDAC	III	Treatment-naïve	Peripheral Blood	
HD1924	PDAC	III	Treatment-naïve	Peripheral Blood	
HD1865	Chronic Pancreatitis			Peripheral Blood	
HD1882	Autoimmune Pancreatitis			Peripheral Blood	
